# A Novel Inflammatory lncRNAs Prognostic Signature for Predicting the Prognosis of Low-Grade Glioma Patients

**DOI:** 10.3389/fgene.2021.697819

**Published:** 2021-08-02

**Authors:** Zijin Xiang, Xueru Chen, Qiaoli Lv, Xiangdong Peng

**Affiliations:** ^1^Department of Pharmacy, The Third Xiangya Hospital, Central South University, Changsha, China; ^2^Jiangxi Key Laboratory of Translational Cancer Research, Jiangxi Cancer Hospital of Nanchang University, Nanchang, China

**Keywords:** glioma, lncRNA, inflammation, immunity, prognosis

## Abstract

**Background:**

As immunotherapy has received attention as new treatments for brain cancer, the role of inflammation in the process of glioma is of particular importance. Increasing studies have further shown that long non-coding RNAs (lncRNAs) are important factors that promote the development of glioma. However, the relationship between inflammation-related lncRNAs and the prognosis of glioma patients remains unclear. The purpose of this study is to construct and validate an inflammation-related lncRNA prognostic signature to predict the prognosis of low-grade glioma patients.

**Methods:**

By downloading and analyzing the gene expression data and clinical information of the Cancer Genome Atlas (TCGA) and Chinese Glioma Genome Atlas (CGGA) patients with low-grade gliomas, we could screen for inflammatory gene-related lncRNAs. Furthermore, through Cox and the Least Absolute Shrinkage and Selection Operator regression analyses, we established a risk model and divided patients into high- and low-risk groups based on the median value of the risk score to analyze the prognosis. In addition, we analyzed the tumor mutation burden (TMB) between the two groups based on somatic mutation data, and explored the difference in copy number variations (CNVs) based on the GISTIC algorithm. Finally, we used the MCPCounter algorithm to study the relationship between the risk model and immune cell infiltration, and used gene set enrichment analysis (GSEA), Gene Ontology (GO), and Kyoto Encyclopedia of Genes and Genomes (KEGG) analyses to explore the enrichment pathways and biological processes of differentially expressed genes between the high- and low-risk groups.

**Results:**

A novel prognostic signature was constructed including 11 inflammatory lncRNAs. This risk model could be an independent prognostic predictor. The patients in the high-risk group had a poor prognosis. There were significant differences in TMB and CNVs for patients in the high- and low-risk groups. In the high-risk group, the immune system was activated more significantly, and the expression of immune checkpoint-related genes was also higher. The GSEA, GO, and KEGG analyses showed that highly expressed genes in the high-risk group were enriched in immune-related processes, while lowly expressed genes were enriched in neuromodulation processes.

**Conclusion:**

The risk model of 11 inflammation-related lncRNAs can serve as a promising prognostic biomarker for low-grade gliomas patients.

## Introduction

Gliomas are the most common subtype of primary brain tumor, accounting for ∼40–50% of primary intracranial malignancy. What is more, gliomas are considered deadly tumors, mainly due to the diffuse infiltration of tumor cells and resistance to radiotherapy and chemotherapy (McNeill, 2016). The World Health Organization (WHO) classifies Grade I and II tumors as low-grade gliomas (LGGs)(Louis et al., 2016). Overall, patients with LGGs have a good prognosis. However, LGGs still have a definite recurrence rate and the potential to increase the grade of malignancy, since invasive gliomas are difficult to completely excise by surgery (Wessels et al., 2003; Duffau and Taillandier, 2015). At present, the diagnosis, monitoring, and treatment of glioma still rely on invasive operations such as biopsy and craniotomy, which can cause serious harm to patients (Hervey-Jumper and Berger, 2016). Therefore, we urgently need a fast and safe method to facilitate a preoperative evaluation of glioma patients. Although the detection of molecular biomarkers has promoted the clinical diagnosis and treatment of glioma, the prediction of individual molecular markers is still defective. We need to develop more and more appropriate molecular models to predict the prognosis of glioma patients.

Inflammation is a positive response of the immune system to infection, trauma, or other stress, and plays an indispensable role in maintaining the health of the body (Sowers et al., 2014). However, a growing body of research evidence indicates that inflammation also plays an important role in promoting the occurrence and development of malignant tumors, including glioma (Colquhoun, 2017; Feng et al., 2019). The contribution of inflammation in glioma progression involves the interaction of multiple pathways such as oxidative stress, interleukin, tumor necrosis factor-α (TNF-α), and pro-inflammatory transcription factors (NF-κB, STAT3)(Mostofa et al., 2017). Currently, numerous reports are suggesting that abnormal epigenetic changes, including DNA methylation, histone modification, chromatin remodeling, and non-coding RNA regulation, also accelerate the transformation of inflammation to cancer (Nallasamy et al., 2018). Long non-coding RNAs (LncRNAs) are a new class of non-coding RNAs that can regulate glioma-related cellular signaling pathways (Peng et al., 2018). In addition, the potential of these lncRNAs as biomarkers and therapeutic targets in glioma therapy is currently being evaluated. For example, LncRNA-135528 inhibits tumor progression by upregulating CXCL10 via the JAK/STAT pathway (Wang et al., 2018), and MALAT1 regulates the inflammatory response of microglia after lipopolysaccharide stimulation (Zhou et al., 2018). However, lncRNAs associated with inflammation have not been fully characterized in studies of glioma. The value of inflammation-related lncRNAs as potential markers remains unclear in gliomas.

In this study, inflammatory lncRNAs were screened based on the RNA sequence data of LGG patients from the Cancer Genome Atlas (TCGA) database. A risk prognostic model was established by Cox regression and Least Absolute Shrinkage and Selection Operator (LASSO) regression analyses. According to the median value of the risk score, patients were divided into low- and high-risk groups. The prognosis of patients in both groups was further analyzed. In addition, the differences in the tumor mutation burden (TMB), copy number variations (CNVs), and immune infiltration between the two groups were evaluated. What is more, the model was validated in the Chinese Glioma Genome Atlas (CGGA) cohort. In summary, the inflammatory lncRNA signature is an independent prognostic factor and potential treatment strategy for glioma patients.

## Materials and Methods

### Data Extraction and Clinical Samples

**FIGURE 1:**
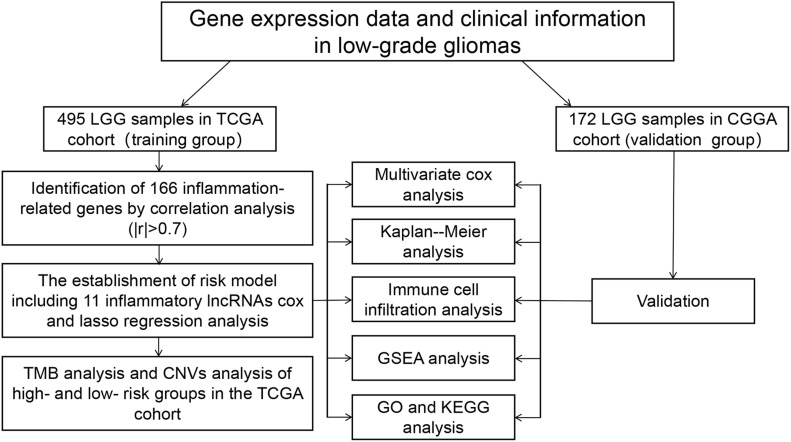
Analysis flow chart.

A flowchart of this study is shown in Figure 1. The RNA-Seq transcriptome profiling data of HTseq-counts type and clinical information related to LGGs were downloaded from the TCGA database.^1^ The data of counts were transformed into TPM for the next analysis. A total of 495 LGG samples were included, excluding the samples with incomplete information. Similarly, WHO I and II samples from the CGGA database^2^ were downloaded, containing 172 cases. Gene Ensembl ID was annotated as Gene Symbol through GENCODE v27. In this study, the data of TCGA were recognized as the training set, and the data of CGGA were recognized as the verification set. What is more, three normal tissue samples and five samples of low-grade glioma patients were obtained from the First Affiliated Hospital of Nanchang University. This study was approved by the Ethics Committee of the First Affiliated Hospital of Nanchang University (No. 2010-015). All patients received informed consent.

### Acquisition of Inflammation-Related lncRNAs

First, inflammation-related genes were obtained in the Gene database from the NCBI.^3^ A total of 2,810 inflammatory genes were included in the study using “(inflammatory) AND “homo sapiens” [porgn: _txid9606]” as filtering conditions (Choe et al., 2021). Next, after extracting the expression data of inflammatory genes mRNA and lncRNA shared by TCGA and CGGA, the correlation between lncRNA and inflammation-related genes in LGGs was analyzed according to the Pearson method (Schober et al., 2018). The lncRNAs with a correlation coefficient of | *r*| > 0.7 were regarded as inflammation-related lncRNAs.

### The Prognostic Modeling by Cox Analysis and LASSO Regression Analysis

In order to further screen the lncRNAs related to the prognosis, according to the univariate Cox regression analysis, all the inflammation-related lncRNAs were performed to correlate with the prognosis of the patient. Subsequently, lncRNAs with adj-*p* values less than 0.0001 were used as candidate lncRNAs to perform lasso penalty with Cox regression analysis for generating inflammation-related lncRNA signature models. According to the lowest Akaike Information Criteria (AIC) value, the best lncRNA prognostic signature was selected for further analysis. The calculation formula used to determine the risk score of each patient based on this prognostic signature model was as follows: risk score = $\sum_{n=1}^{n}C\,o\,e\,f\,\left(i\right)\times x\,\left(i\right)$, where Coef(i) and x(i) represent the estimates of each regression coefficient and expression values of inflammation-related lncRNAs, respectively. Furthermore, patients were divided into high- and low-risk groups using the median risk score as a cutoff. The “survival,” “survminer,” and “survivalROC” R packages were used for survival analysis. Kaplan-Meier survival analysis was used to assess the prognosis between the two groups. To verify the accuracy, the predicted ROC curves of 1–, 3–, and 5-year were established.

### TMB and CNVs Analyses

For the TMB analysis, mutation data were downloaded from the cbioportal database,^4^ and somatic mutation data were visualized in the Mutation Annotation Format (MAF) through the “maftoools” R package (Mayakonda et al., 2018). The TMB value was then estimated by dividing the number of somatic mutations by the total length of the exons. The difference of TMB was further analyzed between the high- and low-risk groups. For the CNVs analysis, the copy number variations of LGGs patients were assessed using the GISTIC algorithm (Mermel et al., 2011), and then, the CNVs differences between the high- and low-risk groups were compared.

### Analysis of Tumor-Infiltrating Immune Cells

The “MCPcounter” R package was used to calculate the abundance of eight immune cells and two stromal cells in the sample to explore the differences in tumor microenvironment under the different groups (Newman et al., 2015; Becht et al., 2016). Furthermore, the expression of immune checkpoint-related genes was analyzed in the high- and low-risk groups.

### Functional Enrichment Analysis

The “clusterProfiler” package was performed for gene set enrichment analysis (GSEA) in the high- and low-risk groups. Furthermore, Gene Ontology (GO) and Kyoto Encyclopedia of Genes and Genomes (KEGG) analyses were carried out on the differentially expressed genes (| log_2_(FC)| > 1.5, *p*-adj < 0.05) between the high- and low-risk groups.

### LncRNAs Expression Analysis

GEPIA^5^ is an online analysis tool for cancer and normal gene expression profiling and integrated analysis. The mRNA expression analysis and prognosis analysis of single-gene in the risk model were performed in GEPIA (Tang et al., 2017). The GEPIA database contains 518 LGG samples and 207 normal samples. What is more, the raw data of the GSE4290 dataset were downloaded from the Gene Expression Omnibus (GEO) database for lncRNA expression. There were 23 normal tissues and 45 LGGs tissues in GSE4290.

### Quantitative Real-Time PCR

Total RNA was extracted from tissues by the Trizol reagent (Invitrogen, Carlsbad, CA, United States). RNA was further reverse-transcripted into cDNA using the Thermo Scientific Revertaid First Strand cDNA Synthesis Kit (Thermo Fisher Scientific, Wilmington, DE, United States). Then, quantitative real-time PCR (qRT-PCR) was performed using the SYBR Green Real-time PCR kit (Takara, Dalian, China). The qPCR primer sequences are listed in Supplementary Table 1.

### Statistical Analysis

All statistical analyses were performed using the R software (version 4.0.4^6^). The expression of single lncRNAs in tumor tissues and normal tissues was performed by the unpaired *t*-test in the GraphPad Prism 7.0 software. *P* < 0.05 was considered statistically significant.

## Results

### Patient Data Processing and Model Building

**FIGURE 2:**
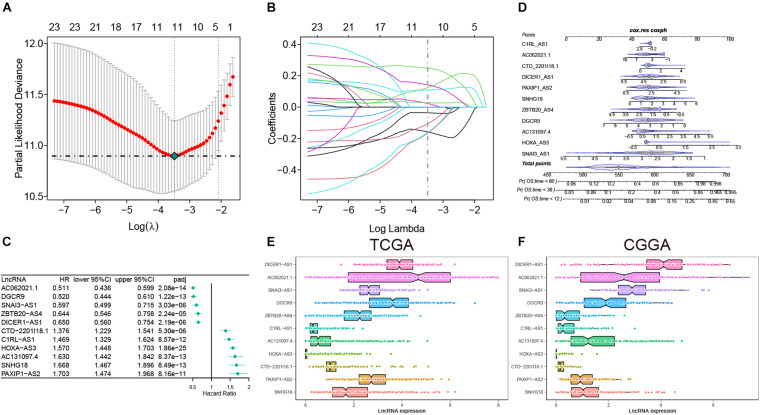
The construction of risk model and identification of inflammation-related lncRNAs. **(A)** Partial likelihood deviation was plotted relative to the logarithm of lambda in 10-fold cross-validation. **(B)** The trajectory graph of each variable. **(C)** The forest plot of univariate Cox regression analysis among 11 lncRNAs. **(D)** Nomogram to predict the 1–, 3–, and 5-year OS for 11 lncRNAs. **(E,F)** The expression of 11 lncRNAs in the TCGA and CGGA cohorts.

In this study, a total of 667 of patients were included and their low-grade glioma gene expression data and related clinical information were analyzed. Among them, 495 were in TCGA and 172 were in CGGA. We summarized the clinical information of the samples in the two databases in Supplementary Table 2, including age, gender, survival status, IDH mutation status, MGMT methylation, and 1p19q codel. In the training set, we screened a total of 166 inflammatory gene-related lncRNAs (| *r*| > 0.7) for further univariate Cox prognostic analysis. Univariate Cox analysis showed that 24 inflammation-related lncRNAs were significantly associated with the prognosis of glioma patients (adj-*p* < 0.0001). Furthermore, 11 lncRNAs were screened and these gene coefficients were obtained to construct a risk model through LASSO and Cox regression analyses (Figures 2A–C). The names and coefficients of 11 lncRNAs are shown in Table 1. A nomogram map was used to show that 11 lncRNAs predicted the 1–, 3–, and 5-year survival rates of glioma patients (Figure 2D). The expression of 11 lncRNAs in the TCGA and CGGA cohorts is illustrated in Figures 2E,F.

**TABLE 1:** Construction of inflammation-related lncRNAs in the risk model.

**gene**	**coef**	**hr**	**low.ci**	**upp.ci**
SNAI3–AS1	–0.1660	0.5971	0.4989	0.7145
ZBTB20–AS4	–0.1627	0.6435	0.5462	0.7581
DGCR9	–0.1586	0.5204	0.4441	0.6100
DICER1–AS1	–0.1063	0.6502	0.5605	0.7542
AC062021.1	–0.0368	0.5110	0.4359	0.5990
CTD-2201I18.1	0.0229	1.3760	1.2289	1.5408
HOXA-AS3	0.0280	1.5701	1.4478	1.7028
C1RL-AS1	0.1089	1.4692	1.3291	1.6239
AC131097.4	0.1248	1.6301	1.4423	1.8423
PAXIP1-AS2	0.1415	1.7031	1.4741	1.9676
SNHG18	0.2477	1.6680	1.4673	1.8961

### The Association Between the Risk Model and Prognosis of Glioma Patients

**FIGURE 3:**
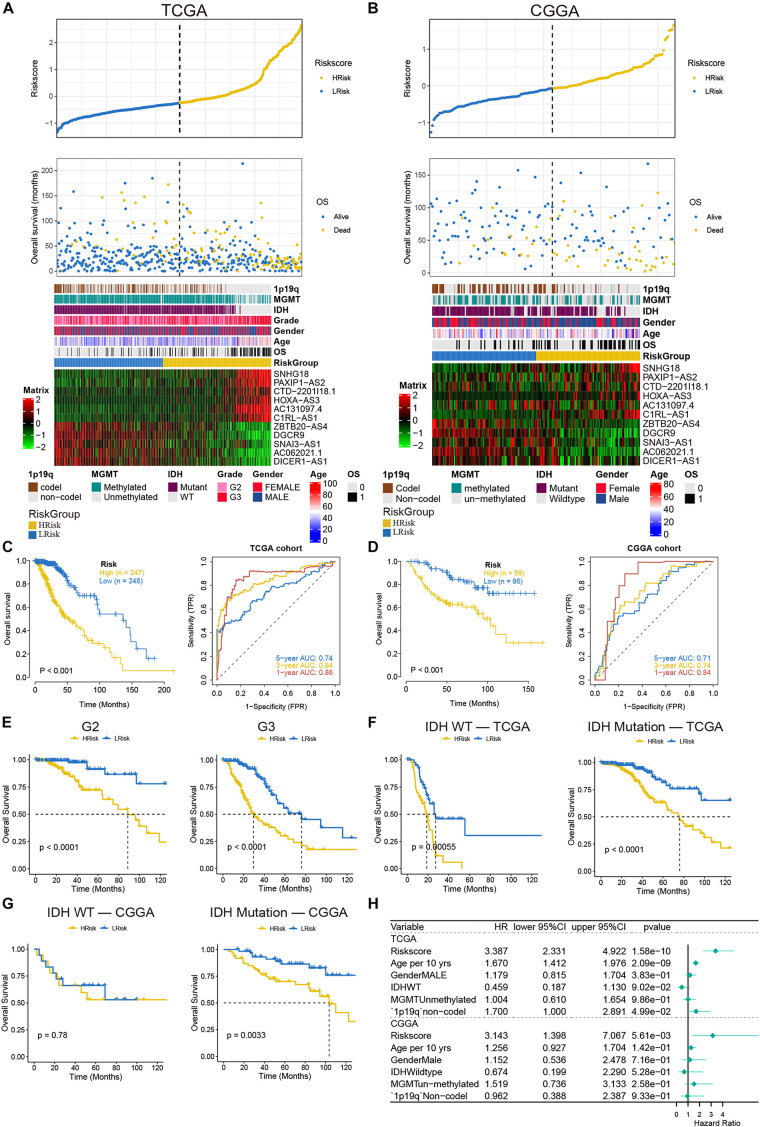
Analysis of prognostic survival of patients with glioma by the risk model. **(A,B)** The risk score value of each sample, the survival status ranked from low to high-risk scores, and the expression heat map of 11 lncRNAs and clinical features in the TCGA **(A)** and CGGA **(B)** cohorts. **(C,D)** Kaplan-Meier curves and ROC curves were drawn by dividing the high- and low-risk groups based on the risk model in the TCGA **(C)** and CGGA **(D)** cohorts. **(E)** Kaplan-Meier curves based on stages in the TCGA cohort. **(F,G)** Kaplan-Meier curves based on IDH status in the TCGA **(F)** and CGGA **(G)** cohorts. **(H)** The forest plot of multivariate Cox regression analysis including the risk model and clinical features.

To verify the effect of the model, the patients of the two cohorts were divided into high- and low-risk groups according to the median value of the risk score. We found that as the risk score increased, the mortality rate gradually increased. In addition, in the high-risk group, IDH mutation rate, MGMT methylation degree, and 1p19q codel rate were significantly reduced, and the expressions of SNHG18, PAXIP1-AS2, CTD-2201I18.1, HOXA-AS3, AC131097.4, and C1RL-AS1 were increased, while the expressions of ZBTB20–AS4, DGCR9, SNAI3–AS1, AC062021.1, and DICER1–AS1 were decreased (Figure 3A). Similar results were also indicated in the validation set (Figure 3B). Next, the overall survival rate results indicated that patients in the low-risk group had a better prognosis than patients in the high-risk group. The ROC curves showed that the AUC values of 1–, 3–, and 5-year were greater than 0.7 in the TCGA and CGGA cohorts (Figures 3C,D). More importantly, in the TCGA cohort, the survival of the low-risk group was still better after the patients were separated by grade (Figure 3E). Considering the IDH mutation factor, the model also predicted that the low score patients in the IDH mutation type had a good prognosis in the two cohorts (Figures 3F,G). The results of multivariate Cox regression analysis demonstrated that the risk model can be independent of other factors as a prognostic indicator, especially in the CGGA cohort (Figure 3H). These results implied that our model had a good predictive ability.

### TMB and CNVs Analyses in the Low- and High-Risk Groups of the TCGA Cohort

**FIGURE 4:**
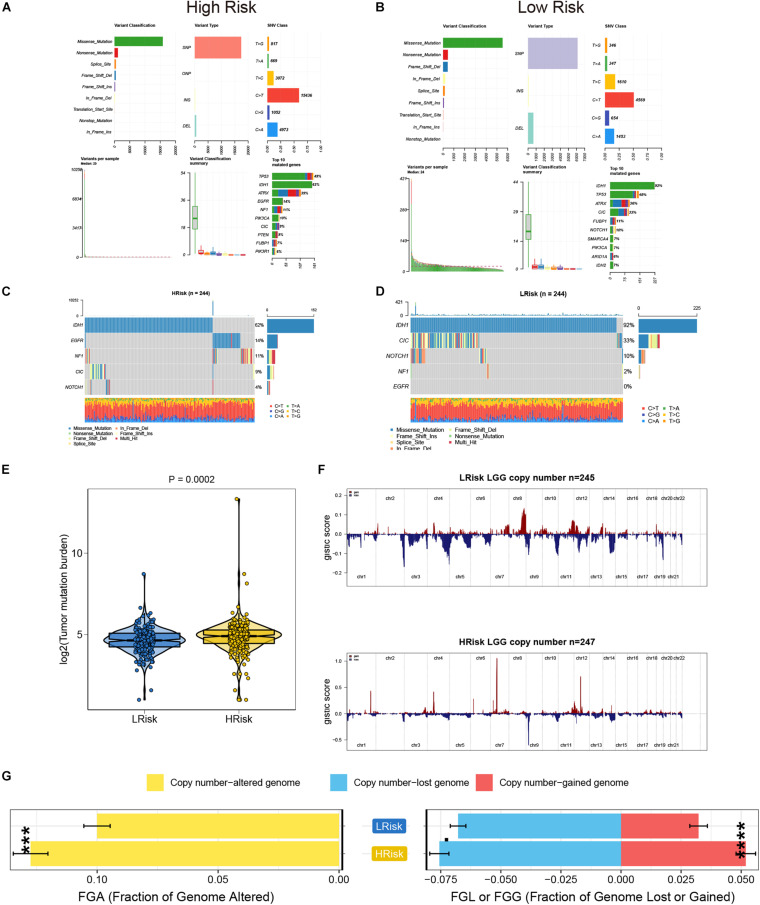
The analysis of TMB and CNVs in the high- and low-risk groups in the TCGA cohort. **(A,B)** Basic information on somatic mutations in glioma patients in the high- and low-risk groups. **(C,D)** The waterfall plot of the mutation profile of a single gene in each glioma sample. **(E)** The differences of TMB in the high- and low-risk groups. **(F)** The amplification and deletion of gene fragments in glioma patients among the two groups. **(G)** The comparison of copy number of the altered genome, lost genome, and gained genome between the high- and low- risk groups. ***p < 0.001, ****p < 0.0001.

We analyzed the somatic mutation data of the TCGA database samples by distinguishing the high- and low-risk groups, and visualized these mutation data. For Variant Classification, missense mutations were obviously common in other mutations. For different Variant Types, SNP was responsible for most of the variants, and single nucleotide variants (SNV) mostly appeared on C > T. Furthermore, we analyzed the specific mutant genes in the two groups, and found that the top 10 mutant genes were slightly different, but mainly TP53, IDH1, and ATRX ranked among the top three (Figures 4A,B). In addition, the mutation details of each glioma sample are shown in the waterfall charts (Figures 4C,D). Moreover, the TMB value of the high-risk group was higher than that of the low-risk group (*P* < 0.05) (Figure 4E). Based on the GISTIC algorithm, we analyzed the CNVs data to identify the genes with obvious amplification or deletion. The distribution of copy number changes in the high- and low-risk groups are presented in Figure 4F. In general, there was a significant difference in the copy number-altered genome between the two groups. The copy numbers of the lost genome and gained genome of the high-risk group were higher than those of the low-risk group (Figure 4G).

### The Risk Model Is Closely Related to Immune Infiltration

**FIGURE 5:**
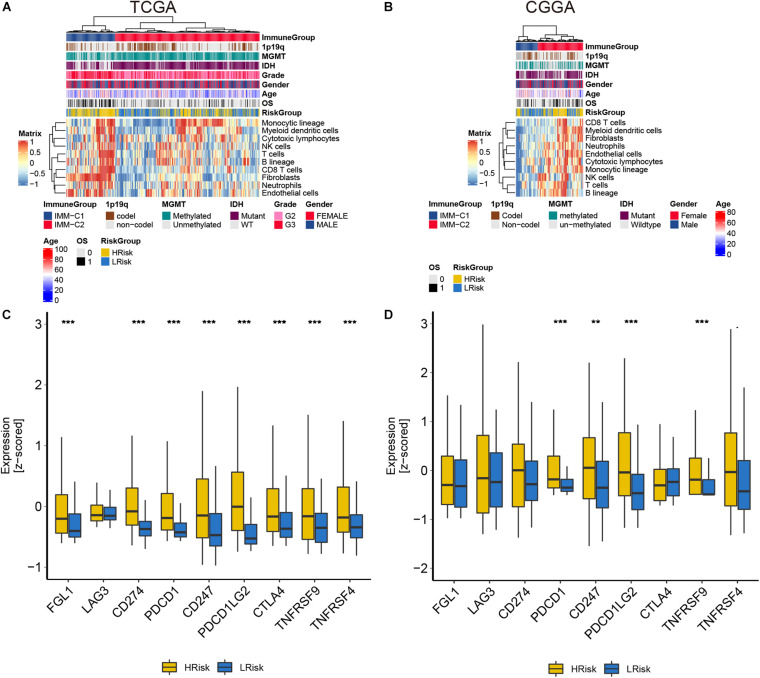
The analysis of tumor immune infiltration in glioma patients in the TCGA and CGGA cohorts. **(A,B)** The heat maps of each immune cell score. **(C,D)** The expression of immune checkpoint-related genes. **p < 0.01, ***p < 0.001.

In the course of disease occurrence, the inflammatory response must be accompanied by the immune response. To further clarify the differences in the immune microenvironment between the high and low-risk groups, we used the MCPcounter algorithm to analyze the composition of tumor-infiltrating immune cells in glioma patients. We found that the CD8 T cells and fibroblasts were significantly enriched in the high-risk group of the two cohorts (Figures 5A,B). Further analysis showed that immune checkpoint-related genes were significantly different between the high- and low-risk groups. The expression levels of these checkpoint-related genes were generally higher in the high-risk group than in the low-risk group (Figures 5C,D). These results implied that patients in the high-risk group had a highly infiltrating immune tumor environment.

### Functional Enrichment Analysis

**FIGURE 6:**
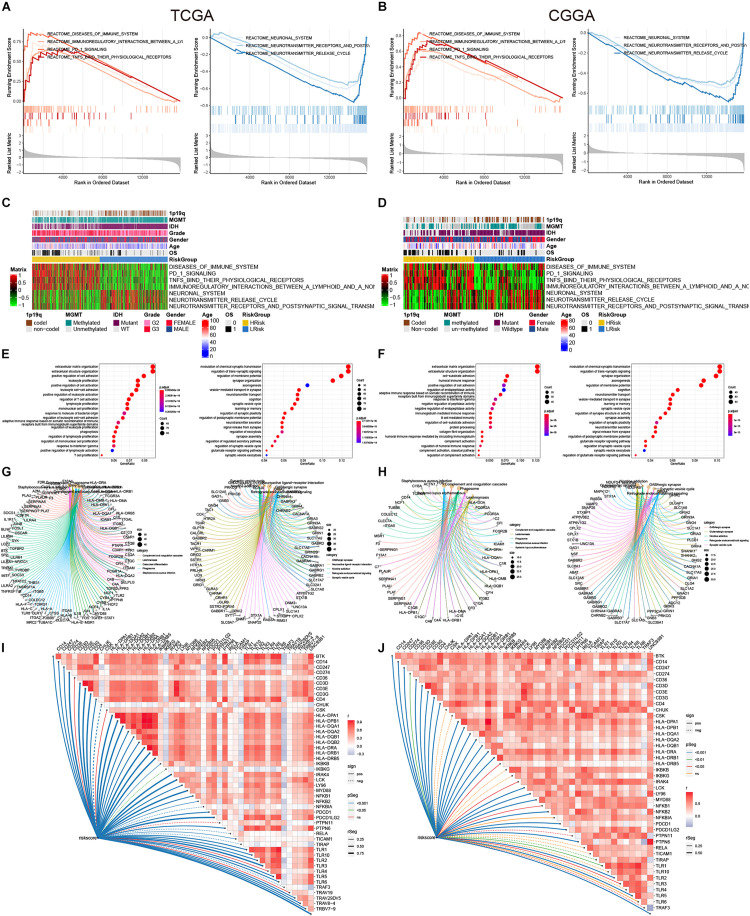
GSEA, GO, and KEGG analysis. **(A,B)** Regulatory pathways of the enrichment analysis in the high-and low-risk groups. **(C,D)** The heat maps of the scores of enrichment pathways in each sample. **(E,F)** GO analysis. **(G,H)** KEGG analysis. **(I,J)** The association between the risk score and some immune-related genes.

In order to further explore the relationship between the risk model and the biological process of glioma, we conducted the GSEA analysis. In the two cohorts, diseases of the immune system, immunoregulatory interactions between a lymphoid and a non-lymphoid cell, PD 1 signaling, and TNFs binding to their physiological receptors were significantly enriched in the high-risk group (Figures 6A,B). While the neuronal system, neurotransmitter receptors, and postsynaptic signal transmission, and neurotransmitter release cycle were significantly enriched in the low-risk group (Figures 6A,B). The scores of these pathways in the samples were displayed in the heat map (Figures 6C,D). At the same time, we further conducted GO and KEGG analyses by screening the differential genes between the high- and low-risk groups [| log_2_(FC)| > 1.5 and adj-*p* < 0.05]. Genes highly expressed in the high-risk group were closely related to immune activation, such as leukocyte proliferation, positive regulation of leukocyte activation, leukocyte cell-cell adhesion, and regulation of T cell activation in the TCGA cohort, while cell-substrate adhesion, humoral immune response, and response to interferon-gamma were observed in the CGGA cohort (Figures 6E,F). However, genes that were low in the high-risk group were mainly related to neuronal regulation in the two cohorts (Figures 6E,F). For the KEGG analysis, upregulated genes were enriched in the phagosome and *Staphylococcus aureus* infection, and downregulated genes were enriched in the GABAergic synapse and Synaptic vesicle cycle (Figures 6G,H). In addition, correlation analysis showed that risk scores were closely related to genes that regulated the immune system (Figures 6I,J). In general, among these differential genes, the relatively highly expressed genes in the high-risk group were mainly involved in the activation of the immune system, while the lowly expressed genes were involved in the regulation of the nervous system.

### Expression Analysis of lncRNA and Confirmation of Prognosis Analysis

**FIGURE 7:**
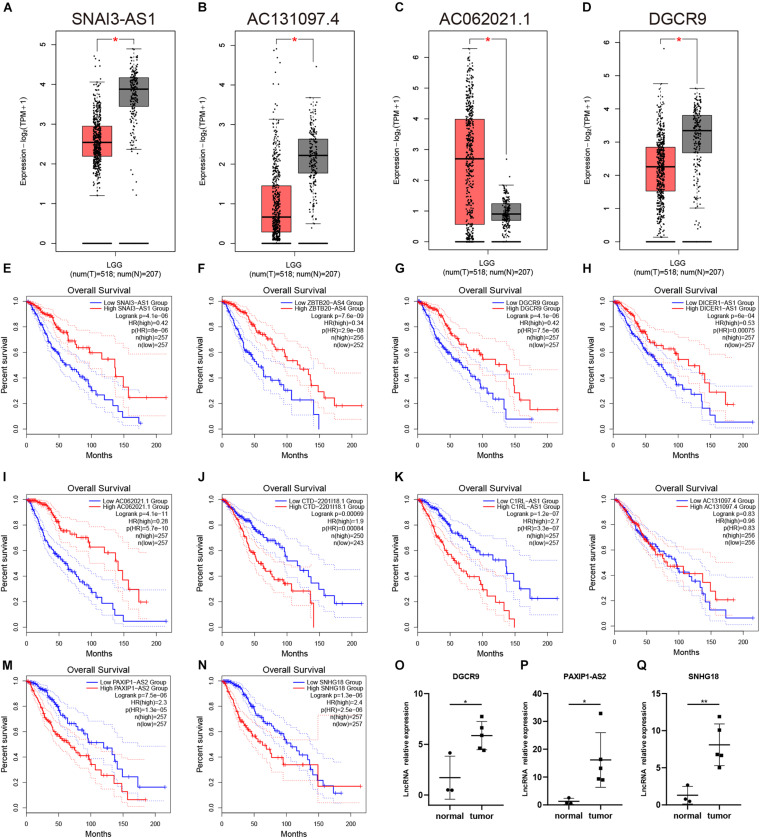
The expression and prognostic analysis of a single lncRNA. **(A–D)** The expressions of SNAI3–AS1, AC131097.4, AC062021.1, and DGCR9 in the GEPIA analysis. **(E–N)** The survival curves of 10 lncRNAs. **(O–Q)** The expressions of DGCR9, PAXIP1-AS2, and SNHG18 in our collected samples. *p < 0.05.

The model we built was based on tumor sample data analysis, so we further wanted to analyze the difference between 11 lncRNAs in the normal samples and tumor samples (Figures 7A–D, Supplementary Figures 1A–N). In the analysis results of the GEPIA database, only the expressions of SNAI3–AS1, AC131097.4, AC062021.1, and DGCR9 were significantly different (*p* < 0.05). Among them, SNAI3–AS1, AC131097.4, and DGCR9 were lowly expressed in tumor tissues and AC062021.1 was highly expressed (Figures 7A–D), while there was no significant difference in the expression level of other lncRNAs. In the GSE4290 dataset, DGCR9 and SNAI3–AS1 were in a lower expression in tumors, while DICER1–AS1 and PAXIP1-AS2 were in a higher expression in tumors. Further survival analysis results were consistent with the univariate Cox analysis results (Figure 2C). The high expression of AC062021.1, DGCR9, SNAI3–AS1, ZBTB20–AS4, and DICER1–AS1 was associated with a good prognosis and appeared to serve as protective factors (Figures 7E–I). Other lncRNAs were used as risk factors, except for AC131097.4, which was not statistically significant, and HOXA-AS3, which had not been queried (Figures 7J–N). Patients with a high expression of these genes had poor prognoses. Moreover, we detected the expressions of these lncRNAs in three normal tissues and five low-grade gliomas collected. Our results showed that the expressions of DGCR9, PAXIP1-AS2, and SNHG18 were higher in the tumor tissues (*P* < 0.05) (Figures 7O–Q), while the expressions of CTD-2201I18.1, DICER1–AS1, and ZBTB20–AS4 showed no difference (Supplementary Figures 1O–Q).

## Discussion

The incidence of gliomas has shown an increasing trend year by year (Siegel et al., 2020). Although low-grade gliomas have a better prognosis, they tend to have high-grade glioma progression due to malignant biological characteristics such as invasive growth and resistance to radiotherapy and chemotherapy (Wessels et al., 2003; Duffau and Taillandier, 2015). In recent years, tumor markers for low-grade glioma have been shown to have a high predictive value in terms of prognosis and treatment. These molecular markers include p53 mutation, 1p/19q gene deletion, MGMT promoter methylation, and IDH1 mutation (Louis et al., 2016). However, glioma is a highly heterogeneous tumor type, and individual differences between tumor patients are very obvious, so the prediction of these molecular markers remains limited (Delgado-López et al., 2017). It is of great significance to explore the potential molecular targets of glioma in depth.

Increasing data support the underlying mechanism of the inflammatory microenvironment driving tumorigenesis, especially stomach cancer, colorectal cancer, etc (Lucas et al., 2017; Greten and Grivennikov, 2019; Oya et al., 2020). In addition, some studies also show that the inflammatory chemokine/receptor axis promotes glioma proliferation, metastasis, and new blood vessel formation (Groblewska et al., 2020). Michelson et al. (2016) summarized the inflammation and the malignant transformation of low-grade gliomas into three steps: Initiation, Promotion, and Progression. In general, inflammation may be a contributing factor to glioma progression.

At present, data mining using bioinformatics is now widely used (Lan et al., 2018). Many tumor prognostic models have been developed based on autophagy, glycolysis, inflammatory genes, and other factors (Chen et al., 2017; Wang et al., 2019; Choe et al., 2021). In addition, more and more researchers have discovered that long non-coding RNA is associated with tumor progression, so many new lncRNA-related models based on autophagy, glycolysis, and inflammatory genes are also used to predict the prognosis of tumor patients. Wang et al. (2019) developed a three-gene risk scoring model related to autophagy that can be used as a prognostic biomarker for glioma patients. Choe et al. (2021) integrated the expression of inflammation-related genes and DNA methylation levels to construct a predictive model to analyze the prognosis of colorectal cancer. Sun et al. (2020) constructed an autophagy-related long non-coding RNA prognostic signature for bladder urothelial carcinoma patients. In this study, we used bioinformatics and statistical tools to systematically analyze the prognostic accuracy of inflammation-related lncRNAs in low-grade gliomas.

Here, we used univariate Cox regression, multivariate Cox regression, and LASSO regression analyses to construct a new signature containing 11 inflammation-related lncRNAs based on the TCGA and CGGA data from low-grade glioma patients, which can be used as independent predictions factor. This model can accurately predict the prognosis of patients where patients with high-risk scores have a poor prognosis. This predictive ability was also demonstrated in the CGGA validation set and applied to patients classified by grade and IDH status. Furthermore, we found differences in the analyses of TMB and CNVs between patients with high and low scores. The high-risk group had higher TMB and CNVs. For the immune cell infiltration analysis, the proportion of immune T cells and B cells in the high-risk group was higher. More importantly, immune checkpoint-related genes were highly expressed in the high-risk group. The analysis results of GSEA, GO, and KEGG related to the model suggested that the highly expressed genes of the high-risk group were mainly enriched in immune-related pathways, such as PD 1 signaling, and low-expressed genes were enriched in neuroregulation pathways, such as neurotransmitter receptors and postsynaptic signal transmission. In conclusion, the risk model can serve as a prognostic predictor independent of other clinical factors. Moreover, the results of functional enrichment analysis suggested that lncRNAs in the model were involved in immune regulation and were expected to become potential targets, which further strengthened the understanding of the potential mechanism of the association between inflammatory response and glioma progression.

In the signature, we also identified the expression and prognostic analyses of 11 lncRNAs. SNAI3–AS1, AC131097.4, AC062021.1, and DGCR9 were significantly differentially expressed in cancer and paracancer from TCGA, and all lncRNAs could well predict the prognosis of patients as protective or risk factors. Among these lncRNAs, the function of ZBTB20–AS4, CTD-2201I18.1, AC131097.4, and PAXIP1-AS2 is unclear. However, SNAI3–AS1 has been reported to promote the proliferation and metastasis of HCC cells (Li et al., 2019, 2020); DGCR9 can enhance the proliferation, migration, and glucose uptake of gastric cancer cells (Ni et al., 2018); DICER1–AS1 promotes malignant behavior of osteosarcoma, colorectal cancer, and other tumors (Gu et al., 2018; Afrough et al., 2020; Ma et al., 2020); and LncRNA C1RL-AS1 silencing inhibited the malignant phenotype of gastric cancer cells through the Akt/β-catenin/c-myc pathway (Zhen-Hua et al., 2020). The function of these four lncRNAs in gliomas is unknown. In addition, SNHG18 promoted radiological resistance of glioma by inhibiting semaphore 5A and promoted glioma cell movement by disrupting α-enolase nucleoplasmic transport (Zheng et al., 2016, 2019). HOXA-AS3 is a risk factor in a variety of tumors, including glioma (Chen et al., 2020). AC062021.1 is involved in the formation of glycolysis-related lncRNA signature that predict the survival in patients with diffuse glioma (Wang et al., 2020).

Although our model has a good predictive effect in the cohorts of TCGA and CGGA, there are still many shortcomings. We should expand the sample to observe the expression of these lncRNAs in normal and tumor tissues. In addition, the functional mechanism of lncRNAs in glioma in the model has not been thoroughly explored. In addition, the effectiveness of the model in clinical practice is unknown, and we intend to undertake further research in the future.

In summary, we first constructed a lncRNA model based on inflammatory genes to predict the prognosis of low-grade glioma patients. The model can be used as a potential prognostic index for glioma patients. In addition, these lncRNAs in the model are expected to be the targets of further glioma therapy.

## Data Availability Statement

The original contributions presented in the study are included in the article/Supplementary Material, further inquiries can be directed to the corresponding author.

## Ethics Statement

The studies involving human participants were reviewed and approved by the Ethics Committee of the First Affiliated Hospital of Nanchang University. The patients/participants provided their written informed consent to participate in this study.

## Author Contributions

XP, ZX, and XC: conception and design. XP: administrative support. QL: provision of study materials or patients. ZX: collection of data. ZX and XC: data analysis and interpretation. All authors contributed to the manuscript writing and approved final version of the manuscript.

## Conflict of Interest

The authors declare that the research was conducted in the absence of any commercial or financial relationships that could be construed as a potential conflict of interest.

## Publisher’s Note

All claims expressed in this article are solely those of the authors and do not necessarily represent those of their affiliated organizations, or those of the publisher, the editors and the reviewers. Any product that may be evaluated in this article, or claim that may be made by its manufacturer, is not guaranteed or endorsed by the publisher.

## Footnotes

1
https://cancergenome.nih.gov/

2
http://www.cgga.org.cn/

3 www.ncbi.nlm.nih.gov/gene (accessed on March 15, 2021)

4
http://www.cbioportal.org/

5
http://gepia.cancer-pku.cn/index.html

6
https://www.r-project.org/

## Supplementary Material

Supplementary Figure 1 The expression of a single lncRNA. **(A–G)** The expressions of PAXIP1-AS2, HOXA-AS3, CTD-2201I18.1, DICER1–AS1, ZBTB20–AS4, SNHG18, and C1RL-AS1 in the GEPIA analysis. **(H–N)** The expressions of DGCR9, SNAI3–AS1, DICER1–AS1, C1RL-AS1, HOXA-AS3, SNHG18, and PAXIP1-AS2 in the GSE4290 dataset. **(O–Q)** The expressions of CTD-2201I18.1, DICER1–AS1, and ZBTB20–AS4 in our collected samples. ****p* < 0.001.

## References

[B1] Afrough H. Ghafouri-Fard S. Yousefi H. Pakzad P. Kholghi Oskooei V. Taheri M. (2020). DICER-AS1 lncRNA: a putative culprit in the pathogenesis of gastric cancer. *Exp. Mol. Pathol.* 116:104490. 10.1016/j.yexmp.2020.104490 32663487

[B2] Becht E. Giraldo N. A. Lacroix L. Buttard B. Elarouci N. Petitprez F. (2016). Estimating the population abundance of tissue-infiltrating immune and stromal cell populations using gene expression. *Genome Biol.* 17:218. 10.1186/s13059-016-1070-5 27765066 PMC5073889

[B3] Chen C. Shi Y. Li Y. He Z. C. Zhou K. Zhang X. N. (2017). A glycolysis-based ten-gene signature correlates with the clinical outcome, molecular subtype and IDH1 mutation in glioblastoma. *J. Genet. Genomics* 44 519–530. 10.1016/j.jgg.2017.05.007 29169920

[B4] Chen W. Li Q. Zhang G. Wang H. Zhu Z. Chen L. (2020). LncRNA HOXA-AS3 promotes the malignancy of glioblastoma through regulating miR-455-5p/USP3 axis. *J. Cell Mol. Med.* 24 11755–11767. 10.1111/jcmm.15788 32918360 PMC7579690

[B5] Choe E. K. Lee S. Kim S. Y. Shivakumar M. Park K. J. Chai Y. J. (2021). Prognostic effect of inflammatory genes on stage I-III colorectal cancer-integrative analysis of tcga data. *Cancers (Basel)* 13:751. 10.3390/cancers13040751 33670198 PMC7916934

[B6] Colquhoun A. (2017). Cell biology-metabolic crosstalk in glioma. *Int. J. Biochem. Cell Biol.* 89 171–181. 10.1016/j.biocel.2017.05.022 28549626

[B7] Delgado-López P. D. Corrales-García E. M. Martino J. Lastra-Aras E. Dueñas-Polo M. T. (2017). Diffuse low-grade glioma: a review on the new molecular classification, natural history and current management strategies. *Clin. Transl. Oncol.* 19 931–944. 10.1007/s12094-017-1631-4 28255650

[B8] Duffau H. Taillandier L. (2015). New concepts in the management of diffuse low-grade glioma: proposal of a multistage and individualized therapeutic approach. *Neuro. Oncol.* 17 332–342. 10.1093/neuonc/nou153 25087230 PMC4483091

[B9] Feng Y. Wang J. Tan D. Cheng P. Wu A. (2019). Relationship between circulating inflammatory factors and glioma risk and prognosis: a meta-analysis. *Cancer Med.* 8 7454–7468. 10.1002/cam4.2585 31599129 PMC6885890

[B10] Greten F. R. Grivennikov S. I. (2019). Inflammation and cancer: triggers, mechanisms, and consequences. *Immunity* 51 27–41. 10.1016/j.immuni.2019.06.025 31315034 PMC6831096

[B11] Groblewska M. Litman-Zawadzka A. Mroczko B. (2020). The role of selected chemokines and their receptors in the development of gliomas. *Int. J. Mol. Sci.* 21:3704. 10.3390/ijms21103704 32456359 PMC7279280

[B12] Gu Z. Hou Z. Zheng L. Wang X. Wu L. Zhang C. (2018). LncRNA DICER1–AS1 promotes the proliferation, invasion and autophagy of osteosarcoma cells via miR-30b/ATG5. *Biomed. Pharmacother.* 104 110–118. 10.1016/j.biopha.2018.04.193 29772430

[B13] Hervey-Jumper S. L. Berger M. S. (2016). Maximizing safe resection of low- and high-grade glioma. *J. Neurooncol.* 130 269–282. 10.1007/s11060-016-2110-4 27174197

[B14] Lan K. Wang D. T. Fong S. Liu L. S. Wong K. K. L. Dey N. (2018). A survey of data mining and deep learning in bioinformatics. *J. Med. Syst.* 42:139. 10.1007/s10916-018-1003-9 29956014

[B15] Li Y. Guo D. Lu G. Mohiuddin Chowdhury A. T. M. Zhang D. Ren M. (2020). LncRNA SNAI3–AS1 promotes PEG10-mediated proliferation and metastasis via decoying of miR-27a-3p and miR-34a-5p in hepatocellular carcinoma. *Cell Death Dis.* 11:685. 10.1038/s41419-020-02840-z 32826862 PMC7442791

[B16] Li Y. Guo D. Ren M. Zhao Y. Wang X. Chen Y. (2019). Long non-coding RNA SNAI3–AS1 promotes the proliferation and metastasis of hepatocellular carcinoma by regulating the UPF1/Smad7 signalling pathway. *J. Cell Mol. Med.* 23 6271–6282. 10.1111/jcmm.14513 31264769 PMC6714236

[B17] Louis D. N. Perry A. Reifenberger G. von Deimling A. Figarella-Branger D. Cavenee W. K. (2016). The 2016 World Health Organization classification of tumors of the central nervous system: a summary. *Acta Neuropathol.* 131 803–820. 10.1007/s00401-016-1545-1 27157931

[B18] Lucas C. Barnich N. Nguyen H. T. T. (2017). Microbiota, inflammation and colorectal cancer. *Int. J. Mol. Sci.* 18:1310. 10.3390/ijms18061310 28632155 PMC5486131

[B19] Ma C. Ma N. Qin L. Miao C. Luo M. Liu S. (2020). DICER1–AS1 promotes the malignant behaviors of colorectal cancer cells by regulating miR-296-5p/STAT3 axis. *Cancer Manag. Res.* 12 10035–10046. 10.2147/cmar.S252786 33116860 PMC7568600

[B20] Mayakonda A. Lin D. C. Assenov Y. Plass C. Koeffler H. P. (2018). Maftools: efficient and comprehensive analysis of somatic variants in cancer. *Genome Res.* 28 1747–1756. 10.1101/gr.239244.118 30341162 PMC6211645

[B21] McNeill K. A. (2016). Epidemiology of brain tumors. *Neurol. Clin.* 34 981–998. 10.1016/j.ncl.2016.06.014 27720005

[B22] Mermel C. H. Schumacher S. E. Hill B. Meyerson M. L. Beroukhim R. Getz G. (2011). GISTIC2.0 facilitates sensitive and confident localization of the targets of focal somatic copy-number alteration in human cancers. *Genome Biol.* 12:R41. 10.1186/gb-2011-12-4-r41 21527027 PMC3218867

[B23] Michelson N. Rincon-Torroella J. Quiñones-Hinojosa A. Greenfield J. P. (2016). Exploring the role of inflammation in the malignant transformation of low-grade gliomas. *J. Neuroimmunol.* 297 132–140. 10.1016/j.jneuroim.2016.05.019 27397086

[B24] Mostofa A. G. Punganuru S. R. Madala H. R. Al-Obaide M. Srivenugopal K. S. (2017). The process and regulatory components of inflammation in brain oncogenesis. *Biomolecules* 7:34. 10.3390/biom7020034 28346397 PMC5485723

[B25] Nallasamy P. Chava S. Verma S. S. Mishra S. Gorantla S. Coulter D. W. (2018). PD-L1, inflammation, non-coding RNAs, and neuroblastoma: immuno-oncology perspective. *Semin. Cancer Biol.* 52(Pt 2) 53–65. 10.1016/j.semcancer.2017.11.009 29196189 PMC5972043

[B26] Newman A. M. Liu C. L. Green M. R. Gentles A. J. Feng W. Xu Y. (2015). Robust enumeration of cell subsets from tissue expression profiles. *Nat. Methods* 12 453–457. 10.1038/nmeth.3337 25822800 PMC4739640

[B27] Ni C. Yang P. Guo J. Ye M. (2018). Role of DiGeorge syndrome critical region gene 9, a long noncoding RNA, in gastric cancer. *Onco. Targets Ther.* 11 2259–2267. 10.2147/ott.S162253 29719408 PMC5914736

[B28] Oya Y. Hayakawa Y. Koike K. (2020). Tumor microenvironment in gastric cancers. *Cancer Sci.* 111 2696–2707. 10.1111/cas.14521 32519436 PMC7419059

[B29] Peng Z. Liu C. Wu M. (2018). New insights into long noncoding RNAs and their roles in glioma. *Mol. Cancer* 17:61. 10.1186/s12943-018-0812-2 29458374 PMC5817731

[B30] Schober P. Boer C. Schwarte L. A. (2018). Correlation coefficients: appropriate use and interpretation. *Anesth. Analg.* 126 1763–1768. 10.1213/ane.0000000000002864 29481436

[B31] Siegel R. L. Miller K. D. Jemal A. (2020). Cancer statistics, 2020. *CA Cancer. J. Clin.* 70 7–30. 10.3322/caac.21590 31912902

[B32] Sowers J. L. Johnson K. M. Conrad C. Patterson J. T. Sowers L. C. (2014). The role of inflammation in brain cancer. *Adv. Exp. Med. Biol.* 816 75–105. 10.1007/978-3-0348-0837-8_4 24818720

[B33] Sun Z. Jing C. Xiao C. Li T. (2020). An autophagy-related long non-coding RNA prognostic signature accurately predicts survival outcomes in bladder urothelial carcinoma patients. *Aging (Albany NY)* 12 15624–15637. 10.18632/aging.103718 32805727 PMC7467376

[B34] Tang Z. Li C. Kang B. Gao G. Li C. Zhang Z. (2017). GEPIA: a web server for cancer and normal gene expression profiling and interactive analyses. *Nucleic Acids Res.* 45 W98–W102. 10.1093/nar/gkx247 28407145 PMC5570223

[B35] Wang P. Peng X. Zhang J. Wang Z. Meng J. Cen B. (2018). LncRNA-135528 inhibits tumor progression by up-regulating CXCL10 through the JAK/STAT pathway. *Apoptosis* 23 651–666. 10.1007/s10495-018-1482-7 30232656

[B36] Wang Y. Zhou W. Ma S. Guan X. Zhang D. Peng J. (2020). Identification of a glycolysis-related LncRNA signature to predict survival in diffuse glioma patients. *Front. Oncol.* 10:597877. 10.3389/fonc.2020.597877 33614485 PMC7892596

[B37] Wang Z. Gao L. Guo X. Feng C. Lian W. Deng K. (2019). Development and validation of a nomogram with an autophagy-related gene signature for predicting survival in patients with glioblastoma. *Aging (Albany NY)* 11 12246–12269. 10.18632/aging.102566 31844032 PMC6949068

[B38] Wessels P. H. Weber W. E. Raven G. Ramaekers F. C. Hopman A. H. Twijnstra A. (2003). Supratentorial grade II astrocytoma: biological features and clinical course. *Lancet Neurol.* 2 395–403. 10.1016/s1474-4422(03)00434-4 12849117

[B39] Zheng R. Yao Q. Li X. Xu B. (2019). Long noncoding ribonucleic acid snhg18 promotes glioma cell motility via disruption of α-enolase nucleocytoplasmic transport. *Front. Genet.* 10:1140. 10.3389/fgene.2019.01140 31798634 PMC6865306

[B40] Zheng R. Yao Q. Ren C. Liu Y. Yang H. Xie G. (2016). Upregulation of long noncoding rna small nucleolar RNA host gene 18 promotes radioresistance of glioma by repressing semaphorin 5A. *Int. J. Radiat. Oncol. Biol. Phys.* 96 877–887. 10.1016/j.ijrobp.2016.07.036 27788958

[B41] Zhen-Hua W. Yi-Wei G. Li-Qin Z. Jie-Yun Z. Zhe G. Wei-Jian G. (2020). Silencing of LncRNA C1RL-AS1 suppresses the malignant phenotype in gastric cancer cells via the AKT/β-Catenin/c-Myc pathway. *Front. Oncol.* 10:1508. 10.3389/fonc.2020.01508 32983994 PMC7492601

[B42] Zhou H. J. Wang L. Q. Wang D. B. Yu J. B. Zhu Y. Xu Q. S. (2018). Long noncoding RNA MALAT1 contributes to inflammatory response of microglia following spinal cord injury via the modulation of a miR-199b/IKKβ/NF-κB signaling pathway. *Am. J. Physiol. Cell Physiol.* 315 C52–C61. 10.1152/ajpcell.00278.2017 29631367

