# Supplementary figures and images for: A Novel Inflammatory lncRNAs Prognostic Signature for Predicting the Prognosis of Low-Grade Glioma Patients
Source: Front Genet. 2021 Aug 2;12:697819. doi: 10.3389/fgene.2021.697819 (PMC8365518; doi:10.3389/fgene.2021.697819)

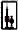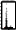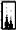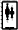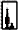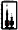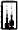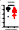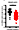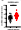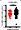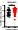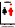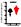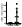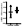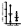

Supplement: Supplementary Figure 1 — The expression of a single lncRNA. (A–G) The expressions of PAXIP1-AS2, HOXA-AS3, CTD-2201I18.1, DICER1–AS1, ZBTB20–AS4, SNHG18, and C1RL-AS1 in the GEPIA analysis. (H–N) The expressions of DGCR9, SNAI3–AS1, DICER1–AS1, C1RL-AS1, HOXA-AS3, SNHG18, and PAXIP1-AS2 in the GSE4290 dataset. (O–Q) The expressions of CTD-2201I18.1, DICER1–AS1, and ZBTB20–AS4 in our collected samples. ***p < 0.001. [file Image_1.PDF]
