# Supplementary material for: A Novel Inflammatory lncRNAs Prognostic Signature for Predicting the Prognosis of Low-Grade Glioma Patients
Source: Front Genet. 2021 Aug 2;12:697819. doi: 10.3389/fgene.2021.697819 (PMC8365518; doi:10.3389/fgene.2021.697819)
Supplement: Supplementary file 2 [file Data_Sheet_1.docx]

Table S1 The primer sequences of lncRNAs

| Gene | Primer |
| --- | --- |
| DGCR9 | Forward TAGCATGGCCAGGTATGCAC |
|  | Reverse TGCGAATCCCAAAGCTGTCA |
| ZBTB20-AS4 | Forward CAGGGTGGCTAACCTACAGC |
|  | Reverse AGAGTGGTCTGGACTCTTGGT |
| DICER1-AS1 | Forward CTGATAGGCCGGTTTGCTCC |
|  | Reverse ACTCCTTCAGCTCCAACTTGT |
| CTD-2201I18.1 | Forward CCGTGTTGCTCAGACATTGC |
|  | Reverse AGGCTCATCTTATCGGCCAC |
| PAXIP1-AS2 | Forward TTGAGCAGGTCCGTGTGTTA |
|  | Reverse GTCACACTTGCCACACTTCAC |
| SNHG18 | Forward TGTGGCAGCCCACTCTATTG |
|  | Reverse TGGTGGACTTGAGTGGAAGC |
| GAPDH | Forward TGACTTCAACAGCGACACCCA |
|  | Reverse CACCCTGTTGCTGTAGCCAAA |

Table S2 The statistics of clinical characteristics of TCGA and CGGA samples

|  | TCGA-LGG (n = 495) | CGGA (n = 172) |
| --- | --- | --- |
| Age, yrs [median, IQR] | 41 [33-53] | 40 [33-45] |
| Gender |  |  |
| Male | 274 (55) | 105 (61) |
| Female | 221 (45) | 67 (39) |
| Overall survival |  |  |
| Dead | 124 (25) | 52 (30) |
| Alive | 371 (75) | 120 (70) |
| IDH |  |  |
| Mutated | 400 (81) | 122 (71) |
| Wild | 92 (18) | 36 (21) |
| MGMT |  |  |
| Methylated | 409 (83) | 70 (41) |
| Un-methylated | 86 (17) | 56 (33) |
| 1p19q |  |  |
| Codel | 162 (33) | 54 (31) |
| Non-codel | 333 (67) | 101 (59) |
